# Pontine Warning Syndrome in a 102-Year-Old Independent Woman: A Rare Cause of Recurrent Fluctuating Neurological Deficits

**DOI:** 10.7759/cureus.112701

**Published:** 2026-07-15

**Authors:** Muhammad Azfar Zariq Bin Abu Bakar, Wan Haleena Rusydah Binti Wan Ahmad Shahmirrudin, Jahanzeb Rehan

**Affiliations:** 1 Stroke Medicine, King's Mill Hospital, Sutton-in-Ashfield, GBR; 2 Respiratory Medicine, Nottingham City Hospital, Nottingham, GBR

**Keywords:** brainstem stroke, capsular warning syndrome, cws, fluctuate, fluctuating stroke, pontine stroke, pontine warning syndrome, pws, stroke

## Abstract

Pontine warning syndrome describes recurrent, transient neurological episodes arising from pontine pathology and carries a high risk of subsequent infarction. We report the case of a 102-year-old woman who presented with repeated, stereotyped episodes of unilateral weakness and dysarthria before developing a confirmed pontine infarct. Early CT imaging was normal, and the diagnosis was established only after MRI demonstrated an acute brainstem lesion. Her clinical course reflects previously described patterns of fluctuating deficits linked to pontine perforator disease, intermittent hypoperfusion, and sensitivity to blood pressure changes. She was treated with dual antiplatelet therapy and made a complete recovery. This case highlights the importance of recognising pontine warning syndrome promptly, as early localisation guides imaging strategy, monitoring, and management.

## Introduction

Pontine warning syndrome describes recurrent, transient neurological episodes arising from pontine pathology. The term was introduced by Saposnik and colleagues to describe repeated, stereotyped motor, sensory, dysarthric, or ocular symptoms that precede a pontine infarct and signal a high risk of deterioration [1]. Although this pattern resembles capsular warning syndrome, several reports have shown that similar stuttering episodes may originate from the pons rather than the internal capsule [2,3]. Early CT imaging is often normal in these cases, and MRI is required to confirm the diagnosis [2,4]. We describe an older woman with recurrent transient symptoms localising to the pons, highlighting the importance of recognising this presentation promptly.

## Case presentation

A 102-year-old White British woman, previously fully independent (modified Rankin Scale (mRS) 0) with a clinical frailty score of 3, developed a sudden onset of slurred speech and left-sided weakness early in the morning around 5:00 am [5,6]. Her initial National Institutes of Health Stroke Scale (NIHSS) score was 14 [7]. Although her symptoms partially improved, her family later noticed that neurological deficits persisted.

**Figure 1:**
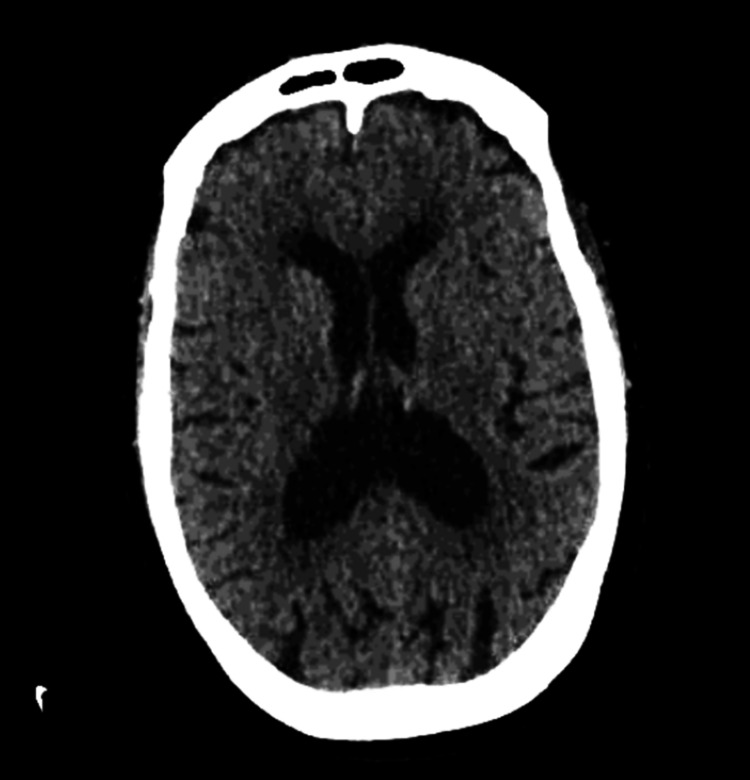
CT head showing no acute intracranial abnormalities

**Figure 2:**
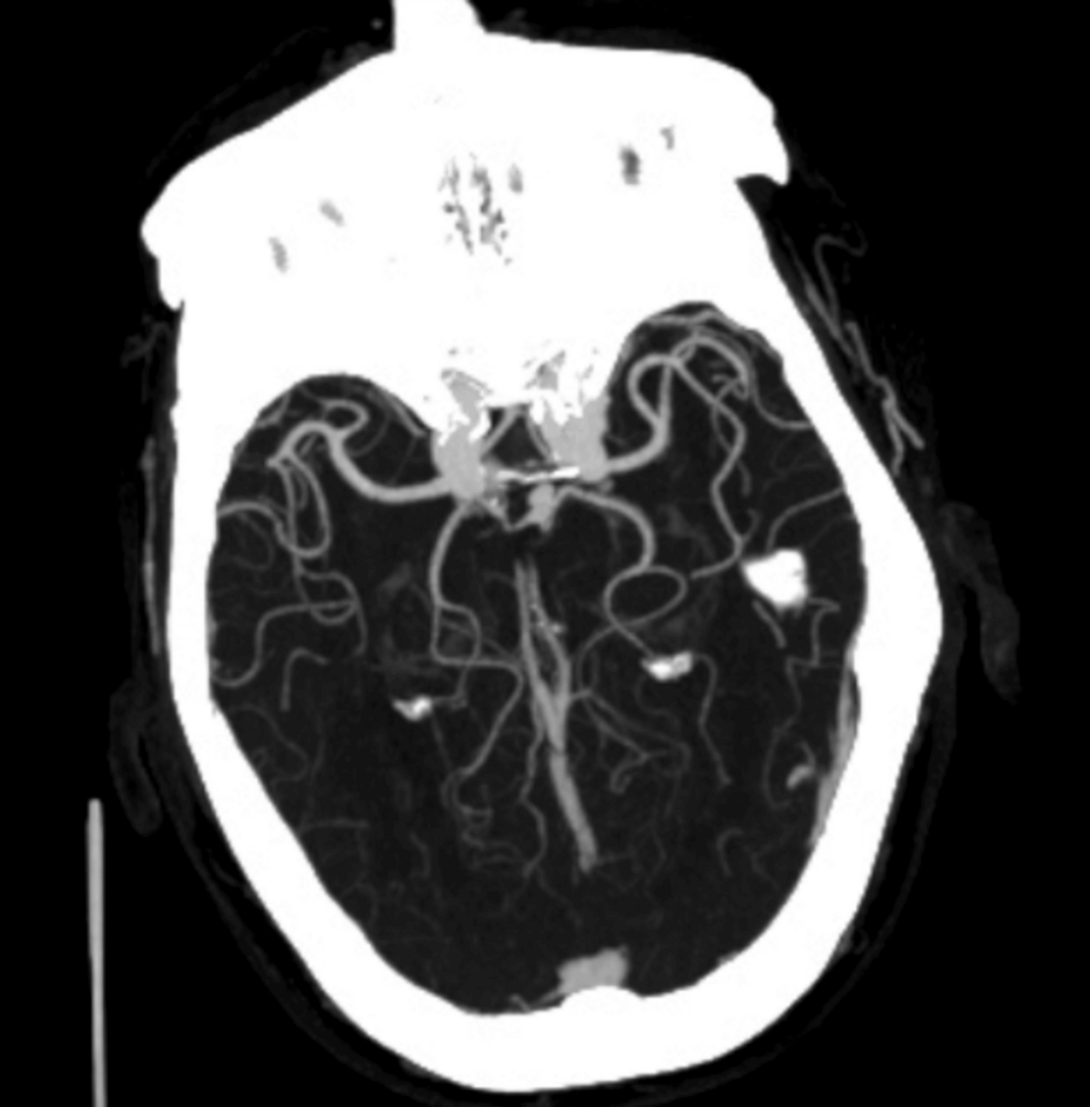
CT angiogram showing patent intracranial arteries

Her relevant medical history included hypertension. She was a lifelong non‑smoker and a teetotal individual. She arrived at the hospital on the same day at 12:00 pm, and on admission, she had systolic blood pressure of 192 mmHg with mild to moderate sensory loss, mild to moderate aphasia, and drift in the left upper and lower limbs, giving an NIHSS score of 4. Cranial nerve examination was normal, and gait could not be assessed at that time. Non-contrast head CT and CT angiography showed no acute abnormality, and patent intracranial arteries were identified (Figures 1-2). Please note that in Figure 2, the appearance interpreted as vertebrobasilar stenosis is more consistent with the level of the imaging slice rather than true luminal narrowing. The basilar artery is clearly visualised, and the apparent tapering occurs just above the vertebral confluence, before both vertebral arteries fully enter the plane.

She was commenced on aspirin 300 mg daily. Her symptoms continued to improve, and by early afternoon, only a sensory disturbance in the left arm remained, with an NIHSS score of 1. She was not a candidate for thrombolysis because she presented outside of the thrombolysis window, and her NIHSS score improved significantly on assessment by the stroke team.

The following day, her systolic blood pressure remained high (194 mmHg). Her losartan dose was increased from 75 mg to 100 mg, and amlodipine 5 mg daily was commenced. Shortly afterwards, she experienced another brief episode of worsening left-sided weakness and speech disturbance, which resolved spontaneously after approximately 30 minutes.

**Figure 3:**
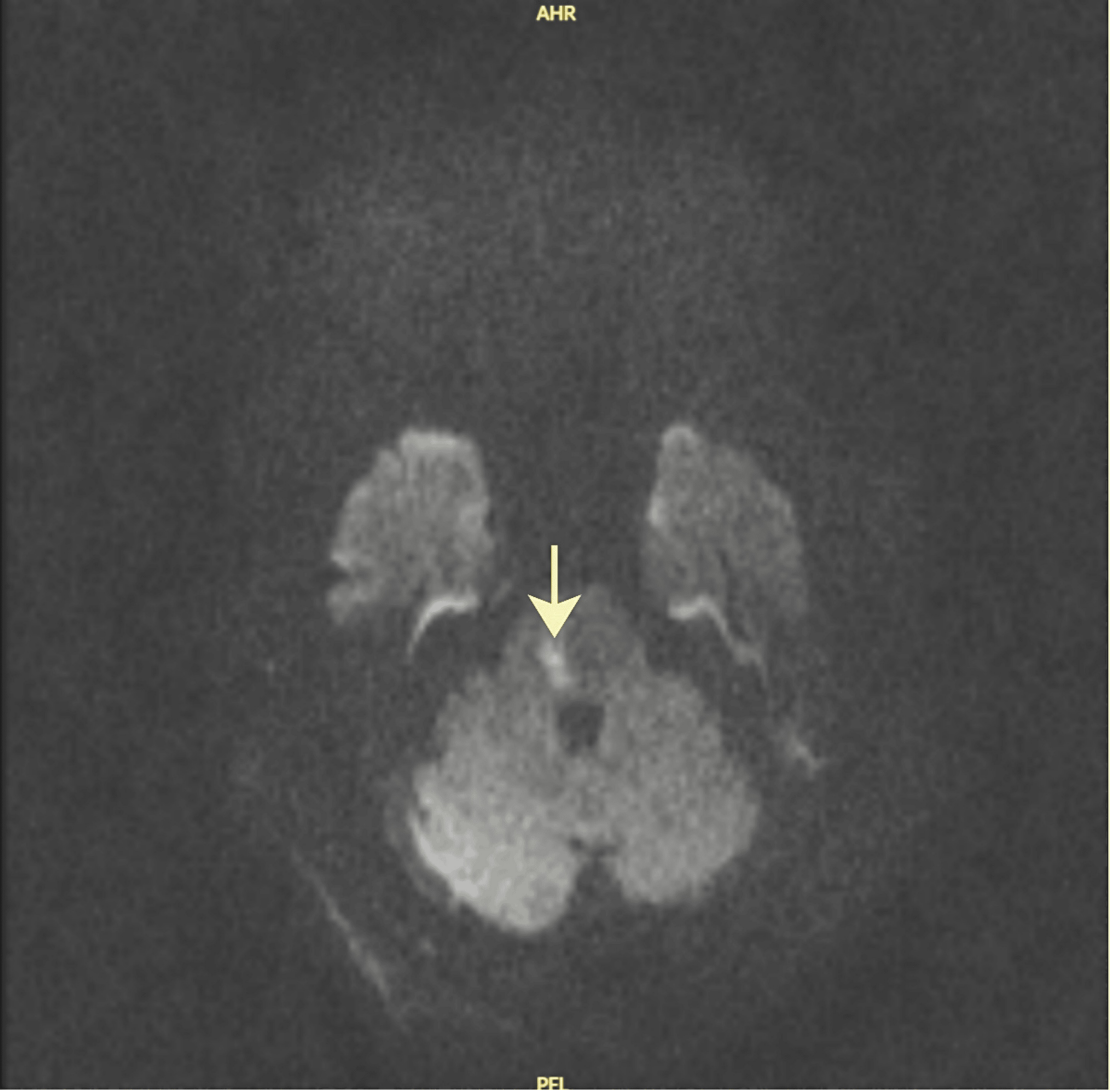
MRI of the head with diffusion-weighted imaging (DWI) sequence demonstrating a small infarct (yellow arrow) in the right pons

She received a loading dose of clopidogrel, and from the following day, she was placed on dual antiplatelet therapy with aspirin 75 mg and clopidogrel 75 mg daily. On the third day of admission, her neurological examination showed normal limb power with only mild residual speech disturbance. MRI of the brain performed that day demonstrated an acute brainstem infarct consistent with a pontine ischaemic stroke (Figure 3).

She was discharged on day 4 on dual antiplatelet therapy. Routine blood tests, including a lipid profile, were performed. Her low-density lipoprotein (LDL) cholesterol was 1.1 mmol/L and non-high-density lipoprotein (non‑HDL) cholesterol 1.7 mmol/L. As her lipid profile was already within the target range, the decision was made to continue her pre‑admission lipid‑lowering therapy of rosuvastatin 5 mg once daily rather than initiate high‑intensity statin therapy [8]. Arrangements for stroke follow‑up and rehabilitation were made. At discharge, her functional status had returned to mRS 0.

## Discussion

This case demonstrates a clinical pattern repeatedly described in reports of capsular and pontine warning syndromes: recurrent, stereotyped, transient neurological episodes that occur over a short period before the development of a fixed deficit. Such fluctuation is a recognised feature of lacunar transient ischaemic attacks and is well documented in capsular warning syndrome, where patients experience multiple near-identical episodes within hours to days [3,9]. Similar stuttering episodes have been reported in pontine warning syndrome, where three or more transient events preceded a definitive pontine infarct [1-4].

The clinical presentation in this case, with recurrent unilateral weakness and dysarthria in the absence of cortical signs, is consistent with a lacunar pattern linked to small-vessel and perforator disease, as described in reports of pontine infarction [1,10]. Several studies emphasise that warning syndromes may arise from the paramedian pons, where single perforating branches of the basilar artery supply the corticospinal tract and related motor pathways [2,3]. This localisation explains how transient motor and dysarthric episodes can occur without cortical features and supports a mechanism centred on pontine perforator involvement.

Huang et al. described how repetitive transient deficits in pontine warning syndrome are most likely driven by intermittent hypoperfusion within the vascular territory of a terminal penetrating artery, caused by branch-related disease rather than lipohyalinosis [2]. Saposnik et al. similarly reported fluctuating symptoms arising from involvement of a single basilar perforator, supporting a mechanism centred on branch pathology [1]. In contrast, Ground and colleagues emphasised that small vessel disease in the pons is strongly associated with hypertension, which promotes lipohyalinosis and increases the susceptibility of perforating arteries to ischaemia [10].

Imaging findings in warning syndromes are frequently subtle in the early phase. In the multicentre cohort reported by He et al., cranial CT was normal on admission in all patients, and infarction was detected only on MRI performed later [9]. Algahtani et al. described a similar pattern, with an initial MRI that appeared normal and a subsequent MRI that demonstrated a pontine infarct [4]. Saposnik et al. also reported normal CT, CT angiography, and CT perfusion despite clear clinical localisation, with MRI later confirming a paramedian pontine infarct [1]. These observations highlight the importance of repeat MRI when symptoms fluctuate despite normal early imaging and support the need for careful clinical localisation in posterior circulation events [10].

Management remains an area of uncertainty. He et al. found no significant difference in outcomes between thrombolysis, single antiplatelet therapy, and dual antiplatelet therapy [9]. Both Ground et al. and Algahtani et al. described the use of dual antiplatelet therapy in pontine infarction, reflecting its common adoption in clinical practice [4,10]. Nadarajan and Adesina initially commenced single antiplatelet therapy, but deterioration prompted escalation to dual antiplatelet treatment [3]. Saposnik et al. reported that thrombolysis appears safe in selected patients with warning syndromes, although their patient did not demonstrate neurological improvement following treatment [1].

Blood pressure management is also complex. Huang et al. cautioned that lowering blood pressure may worsen perfusion in pontine warning syndrome, and Nadarajan and Adesina described deterioration after antihypertensive administration in a similar context [2,3]. A similar pattern was observed in our patient, who experienced another transient episode shortly after her antihypertensive dose was increased. In our case, antihypertensive therapy was initiated more than 24 hours after the ictus, at a time when the patient’s NIHSS had improved to 1 with no other persisting neurological deficit. Given the persistently elevated systolic pressures (>180 mm Hg) since admission and the near‑complete resolution of symptoms, cautious blood pressure control was considered clinically appropriate [11].

Outcomes in pontine warning syndrome vary widely. Severe persistent deficits have been reported despite treatment [1,2], while other patients show substantial recovery over months [3,4]. He et al. reported that more than 80% of patients with capsular warning syndrome achieve a good functional outcome at three months, despite a high rate of infarction [9]. The complete recovery observed in this case aligns with the more favourable end of this spectrum.

Finally, this case reinforces the growing recognition that recurrent lacunar transient ischaemic attacks do not reliably localise to the internal capsule. Pontine infarcts can produce identical clinical patterns at initial presentation, including pure motor or sensorimotor deficits without cranial nerve involvement, as described in several reports [2,3]. Awareness of this variability is essential, as early localisation influences imaging strategy, monitoring, and treatment decisions.

## Conclusions

Pontine warning syndrome is an important cause of recurrent lacunar transient ischaemic attacks and may closely mimic capsular warning syndrome at initial presentation. Recognition of this pattern is essential, as early localisation influences imaging decisions and guides monitoring for deterioration. MRI is often required to confirm the diagnosis when early CT is normal. Although management strategies vary, dual antiplatelet therapy is frequently used, and caution is advised when adjusting blood pressure in the acute phase. This case adds to the growing evidence that pontine perforator disease can produce fluctuating symptoms and that favourable outcomes are achievable when the condition is identified promptly.
